# Barriers to hepatitis B virus screening of pregnant women in primary healthcare centers in Nigeria: health workers’ perspective

**DOI:** 10.1186/s12875-023-02157-8

**Published:** 2023-10-17

**Authors:** Babayemi O. Olakunde, Daniel A. Adeyinka, Olubunmi A. Olakunde, Hasiya B. Raji, Hidayat B. Yahaya, Olugbengba A. Ijaodola, Clement O. Adesigbin

**Affiliations:** 1 Department of Community Prevention and Care Services, National Agency for the Control of AIDS, Ziguinchor Street, off IBB Way, Wuse Zone 4, Abuja, Nigeria; 2 Center for Translation and Implementation Research, University of Nigeria Nsukka, Enugu, Nigeria; 3 Department of Public Health, National AIDS and STI Control Programme, Federal Ministry of Health, Abuja, Nigeria; 4 Department of Disease Control and Immunization, Ondo State Primary Health Care Development Agency, Akure, Nigeria

**Keywords:** Challenges, Perinatal, Prevention of mother-to-child, Testing, Viral hepatitis

## Abstract

**Background:**

Hepatitis B virus (HBV) screening is an important component of antenatal care for pregnant women in Nigeria. However, the screening rates remain low, particularly at primary healthcare centers (PHCs). The objective of this study was to identify the barriers affecting antenatal HBV screening in PHCs in Nigeria from the perspective of health workers.

**Methods:**

We conducted a survey among 30 health workers from 30 PHCs (one per PHC) across three states (Akwa Ibom, Anambra, and Kaduna) in Nigeria. An open-ended questionnaire was used to obtain written responses on the perceived barriers limiting antenatal HBV screening in PHCs and their recommended solutions to the identified barriers. The data were analyzed using an inductive thematic approach.

**Results:**

The perceived barriers exist at patient, provider and health system levels. They included: lack of test kits, unaffordability of HBV test, shortage of trained personnel, poor awareness among pregnant women, knowledge of HBV among health workers, high cost of antiviral treatment, and unavailability of HBV vaccine. The recommended solutions to the identified barriers were: making test kits and vaccines available and free, creating awareness about HBV, and capacity-building interventions for health workers.

**Conclusions:**

HBV screening of pregnant women attending PHCs in Nigeria appears to be affected by multilevel barriers. As the country continues to work towards eliminating HBV, these highlighted barriers at the patient, provider and health system levels must be addressed through effective and sustainable interventions.

## Background

Globally, Africa has the second highest burden of hepatitis B virus (HBV) after Asia [1]. While the risk of perinatal transmission of HBV is lower in Africa compared to Asia, it remains an important mode of transmission in Africa [2]. Estimates indicate that nearly 370,000 newborns are perinatally infected with HBV, annually [2]. Without interventions, 70–90% of infants born to HBV-infected mothers with high viral load and/or hepatitis B e antigen are at risk of being infected [3, 4]. Prevention of mother-to-child transmission (PMTCT) of HBV is important, given that 80–90% of infections acquired in infancy lead to a chronic infection later in life, as opposed to 5% of infections acquired in adulthood [5, 6].

The availability of effective preventive interventions, including active and passive infant immunization and peripartum antiviral maternal prophylaxis [7], underpins the effort to eliminate HBV as a major public health threat in the African region, with impact targets of reducing incidence of chronic HBV and HBV-related deaths by 90% and 65%, respectively by 2030 [8]. As part of the interventions for the PMTCT of HBV, the World Health Organization recommends early HBV screening for all pregnant women in settings with an HBV prevalence of ≥ 2% [9]. However, as of 2021, only 17 (36%) of the 47 countries in the WHO African region had national policies for antenatal HBV screening [10].

Nigeria has one of the largest burdens of HBV infection in the world [1]. With a prevalence of > 5% among pregnant women [11, 12], the country has adopted routine antenatal HBV screening in line with its plan to eliminate vertical transmission of HBV [13, 14]. However, the screening rates remain suboptimal, particularly in primary healthcare centers (PHCs) [15] where a significant proportion of women in rural areas access antenatal care. In a study that assessed the antenatal HBV screening rate among 2.8 million pregnant women who received antenatal care in health facilities providing PMTCT of HIV services in Nigeria, only 7% of the pregnant women were screened for HBV [16]. Similarly, among 643 pregnant women in Oyo State, Nigeria, a study reported that 20% had ever been screened for HBV infection and 9% had received screening in the index pregnancy [15].

Despite the burden of HBV among pregnant women in Nigeria, there is a paucity of data on the factors affecting antenatal HBV screening. Improving the HBV screening rate among pregnant women will require an understanding of the limiting factors, which are likely to operate at patient, providers, community, and/or the healthcare system levels [17, 18]. The objective of this study was to identify the barriers affecting antenatal HBV screening in PHCs in Nigeria from the perspective of health workers.

## Methods

### Study design

This study was a cross-sectional survey of health workers at PHCs in Nigeria. The survey was conducted between October and November 2022.

### Study sites and participants

**Fig. 1:**
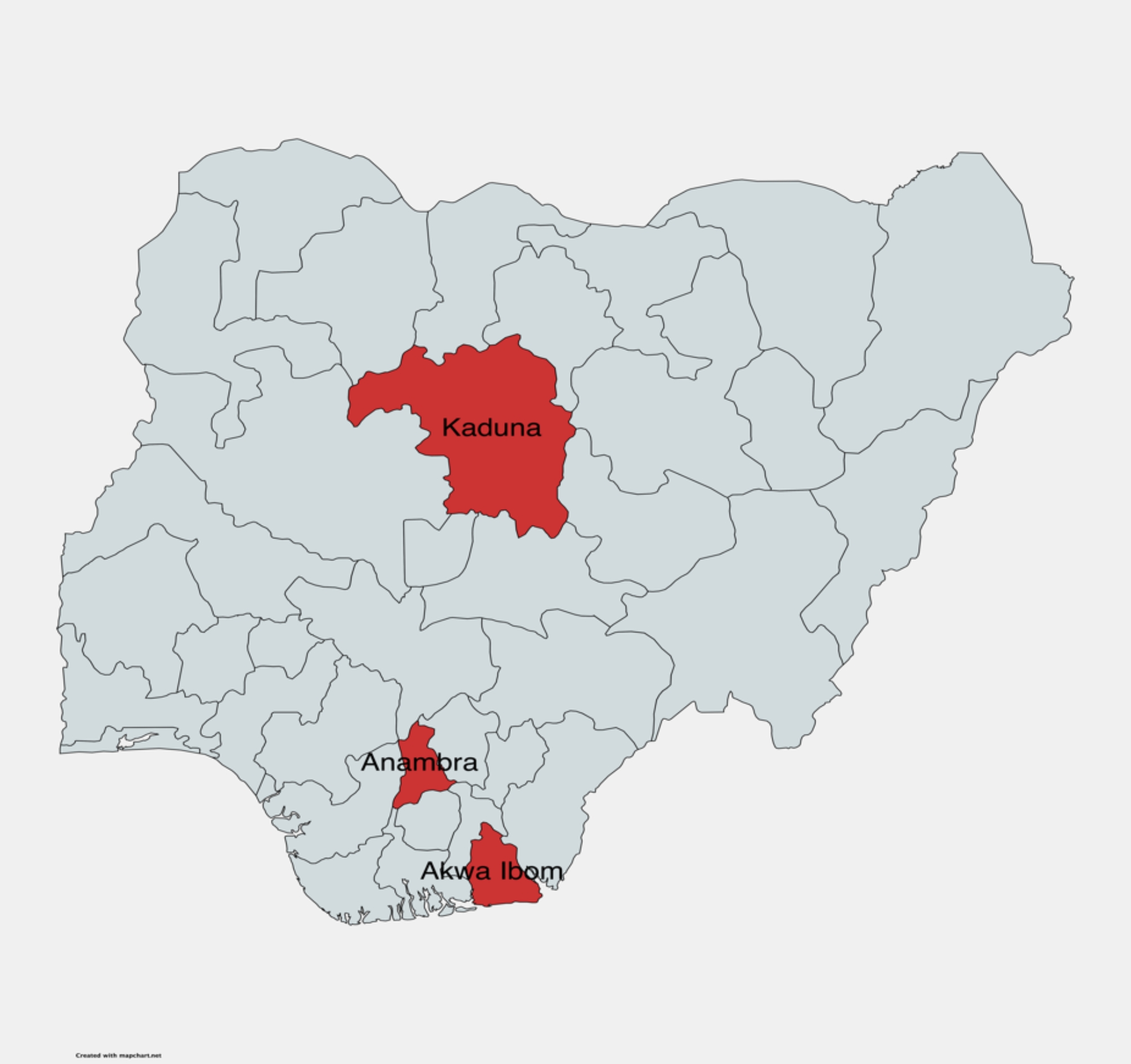
Map of Nigeria showing the study locations

This study leveraged an ongoing Global Fund-funded HIV and Reproductive, Maternal, Newborn, Child, and Adolescent Health Integration pilot project in Akwa Ibom, Anambra, and Kaduna States in Nigeria (Fig. 1). The goal of the pilot project is to improve the coverage of PMTCT of HIV and reproductive, maternal, newborn, child, and adolescent health servicesby strengthening the interface between health facilities and communities. The project is being implemented in 30 PHCs across three rural local government areas (LGAs) in each of the three states. We randomly selected one LGA, comprising 10 PHCs, from each of the three states. Using convenience sampling, we included one health worker from each of the PHCs. Health workers were eligible to participate if they: (i) provided antenatal care, (ii) had been providing the services for at least six months in the facility, and (iii) were willing to complete the survey. Overall, 30 health workers from 30 PHCs were surveyed in the study.

### Study procedure and data collection

With a pen-and-paper personal interview method, data were collected via a self-administered open-ended questionnaire. The questionnaire was pilot tested with three nurses who did not participate in the study. The first section of the questionnaire focused on the demographic characteristics of the respondents. The second section focused on the perceived barriers that affect antenatal HBV screening in PHCs, while the third section was on the recommended interventions to address the identified barriers. The purpose of the study and each section of the questionnaire was explained by the research staff before handing them to the respondents for completion. The respondents were not given any time limit and they were encouraged to write legibly.

### Data analysis

The written responses on the barriers were analyzed using an inductive thematic approach [19]. Codes were assigned to the different segments of the responses to condense or summarize the texts. We searched for patterns in the codes and categorize them into subthemes and then themes. The coding and categorization were initially conducted independently by BOO and OAO and reviewed and harmonized by DAA.

## Results

The sociodemographic characteristics of the respondents are shown in Table 1. The average age was 39.5 years. About 87% of them were females. Most of the respondents were community health extension workers (37%) and about 50% had worked for more than five years in their current health facility.

**Table 1:** Sociodemographic characteristics of health workers (N = 30)

Characteristics	N (%)
Age – Mean (SD)	39.5 years (7.4)
Sex Female Male	26 (87) 4 (13)
Cadre Nurse/midwife Community health officer (CHO) Community health extension worker (CHEW) Junior community health extension worker (JCHEW) Laboratory scientist	10 (33) 7 (23) 11 (37) 1 (3) 1 (3)
Years of service (at the current facility) < 1 1–5 ≥5	6 (20) 9 (30) 15 (50)

### Barriers to HBV testing

The thematic analysis generated three main themes and seven subthemes: (1) Patient-level barriers: (i) Unaffordability of HBV test and (ii) Poor awareness of HBV. (2) Provider-level barrier: (i) Limited knowledge of HBV. (3) Health system-level barriers: (i) Lack of test kits, (ii) Shortage of trained personnel, (iii) High cost of antiviral treatment, and (iv) Unavailability of HBV vaccine.

### Patient-level barriers

#### Unaffordability of HBV test

The cost of HBV screening test was noted as one of the factors limiting the coverage of screening among pregnant women. Many health workers acknowledged that the HBV screening test is not free in their health facilities and that the cost is prohibitive for some pregnant women. Most pregnant women who attend these PHCs in rural areas are of low socioeconomic status and they may not be able to afford the HBV screening test alongside other sundry expenditures required for their antenatal care.

*“Costly. Pregnant women do not have money to carry out the test in addition with other investigation when they come to facility.” (Nurse, Female, 37 years)*.

*“Patient[s] don’t have money to do hepatitis B testing.” (CHEW, Female, 46 years)*.

*“The [HBV] test is not free…pregnant women cannot afford to pay for the test.” (CHEW, Female, 34 years)*.

#### Poor awareness of HBV

Some respondents listed HBV ignorance among pregnant women as a factor that hinders HBV screening. Although the HBV burden is high in Nigeria, many people are not aware of it. Lack of awareness and poor knowledge may negatively affect the demand for HBV screening or acceptance when offered.

*“Ignorance and inadequate knowledge among pregnant women on the subject matter [hepatitis B] is also a barrier.” (Nurse, Female, 40 years)*.

*“Poor knowledge about hepatitis B, that is lack of awareness among the pregnant women.” (CHEW, Female, 51 years)*.

*“Lack of awareness of the patients.” (CHEW, Female, 34 years)*.

### Provider-level barrier

#### Limited knowledge of HBV

Poor awareness of HBV is not limited to pregnant women. Some respondents admitted to inadequate knowledge of HBV among health workers. Such providers may not be knowledgeable about the risk of perinatal transmission and the benefit of screening.

*“…[providers] are not aware. Some of them do not really advice the women on the need to do it.” (Lab scientist, Male, 30 years)*.

*“Inadequate knowledge of healthcare workers” (CHEW, Male, 38 years)*.

### Health systems-level barriers

#### Lack of test kits

A majority of the respondents identified the unavailability of test kits as one of the barriers affecting the HBV screening of pregnant women. If the kits are not available onsite, health workers are not likely to offer the screening test to pregnant women as part of antenatal care. For some health workers, the issue was not only the test kits but other consumables that may be required in carrying out the procedure. Some health workers admitted to recurrent stock out of the test kits.

*“There is no hepatitis B test kit in my facility.” (CHO, Female, 40 years)*.

*“We need enough test kits to carry out the test at all time.” (Nurse, Female, 33 years)*.

*“Inadequate apparatus and lack of reagent for the test.” (Nurse, Female, 30 years)*.

#### Shortage of trained personnel

The health workers also cited insufficient manpower to carry out HBV screening test as a barrier. They noted that their facilities are short-staffed and providing these services may overburden the staff. Moreover, limited capacity of health workers to conduct the screening test was also highlighted as a limiting factor. An untrained health worker may not be able to provide HBV screening services, including, counselling, testing, interpreting the results, and providing appropriate post-screening interventions.

*“Inadequate manpower to provide the services.” (Nurse, Female, 37 years)*.

*“Lack of trained personnel on hepatitis B.” (CHEW, Female, 47 years)*.

*“Inadequate manpower in the facility…one nurse cannot be doing everything.” (Nurse, Female, 40 years)*.

#### High cost of antiviral treatment

Another barrier expressed by a few respondents was the unaffordability of treatment for positive pregnant women. While most PHCs may not be equipped to manage HBVinfection, screen-positive pregnant women are referred to secondary or tertiary health facilities for further investigations and possibly antiviral treatment. The management of HBV is not free in these higher-level facilities and some of the women may not be able to afford it.

*“Patient’s inability to pay for testing and treatment.” (Nurse, Male, 38 years)*.

*“The treatment of hepatitis B is expensive. They cannot afford it if they are positive.” (Nurse, Female, 40 years).*

#### Unavailability of HBV vaccine

Similar to treatment interventions for positive pregnant women after the screening, a few respondents identified the lack of prevention interventions for negative pregnant women as a barrier to HBV screening. For pregnant women who screen negative, they will require a vaccine, which may not be available at the facility.

*“Unavailability of vaccine to give to the women.” (CHEW, Female, 51 years)*.

*“There is no vaccine for negative individuals.” (Nurse, Female, 40 years)*.

### Interventions to improve HBV screening

The respondents also suggested solutions to the barriers affecting antenatal HBV screening. A common intervention recommended by the health workers was the need to make the kits for HBV screening test freely available in the health facilities and provided at no cost to pregnant women. Many of the respondents appropriated this responsibility to the government. Some respondents wrote:

*“Provision of free hepatitis B test kit to the facility will facilitate access.” (CHO, Female, 46 years)*.

*“Government should provide free hepatitis B test kit to the facilities.” (CHO, Female, 40 years)*.

*“Making it [HBV testing] free like HIV testing and TB [Tuberculosis] testing for pregnant women will help.” (CHEW, Female, 51 years)*.

The availability of commodities for HBV screening was not limited to test kits. Some of the respondents also noted the need for vaccines to be widely and freely available, as it may be a motivating factor.

*“Availability of vaccines at all times for pregnant women if negative.” (CHEW, Female, 51 years)*.

*“Hepatitis B vaccine should also be supplied to facilities for pregnant women.” (CHEW, Female, 33 years)*.

Another recommendation by the respondents was creating awareness about HBV and how it can be prevented. This may address the knowledge gap, low-risk perception, and demand for HBV testing services. Recommendations on the approach for awareness creation included the use of community health volunteers or mass campaigns.

*“Provide awareness campaign for the need of every citizen to know his status in respect of HbsAg as it is in the case of HIV.” (CHEW, Male, 40 years)*.

*“The government should engage community volunteers in all communit[ies] to create awareness in other to reduce the virus spread.” (CHEW, Female, 48 years)*.

*“Enlighten the public [on] the need for test and vaccination against the virus.” (CHEW, Female, 28 years)*.

Some respondents also believed that capacity building for health workers will be important in addressing the gaps in HBV screening of pregnant women. Not only will this increase their knowledge, but also the quality of HBV screening services.

*“Organizing training for personnel of hepatitis B testing.” (CHEW, Female, 47 years)*.

*“Adequate training of health workers about the diseases and testing procedure.” (CHEW, Male, 38 years)*.

*“Staff should be trained on the test procedure[,] that is in the use of the test kit.” (Nurse, Female, 44)*.

## Discussion

In this study, we assessed health workers’ perspective on the barriers to HBV screening of pregnant women accessing antenatal care in PHCs in Nigeria. The perceived barriers exist at patient, provider, and health system levels. They included: lack of test kits, unaffordability of HBV test, shortage of trained personnel, poor awareness and knowledge of HBV, and unavailability of treatment and prevention interventions for HBV. The recommended solutions to the identified barriers were: making test kits and vaccines available and free, creating awareness about HBV, and capacity-building interventions for health workers.

Antenatal care is an important entry point to identifying women with HBV and preventing perinatal transmission. However, among the few pregnant women receiving antenatal care in Nigeria, many are not screened for HBV. Our findings suggest that some of the missed opportunities for antenatal HBV screening in PHCs are health system related. The unavailability of basic equipment or health commodities that are needed for the provision of universal health services is a common occurrence in PHCs in Nigeria [20–22]. For example, in a survey of 2480 PHCs from 12 states in Nigeria’s six geopolitical zones, a study reported that only 10.4% of the facilities had HBV vaccine. Moreover, most PHCs, particularly in rural areas, do not have the minimum recommended number of staff and cadre [22–24], thus limiting the scope of work and impacting the quality of services rendered. Although primary healthcare is described as the bedrock of the Nigerian healthcare system, underfunding and poor governance have affected its optimal functionality [20, 25, 26].

The respondents also identified the high costs of the HBV screening test and treatment as limiting factors to providing screening services to pregnant women. As in many African countries [27], HBV screening of pregnant women is not freely available in many health facilities in Nigeria as a result of the limited donor and government support for HBV prevention and control [28]. Even where antenatal care services are free, such screening tests may not be covered, if the kits are not provided by the government. Depending on the location, the HBV screening test in PHCs may cost $3 to $5. In a country where many people live below the poverty line, such amounts may be unaffordable to some individuals [28]. Although the national health insurance scheme covers HBV screening test for pregnant women [29], the health insurance coverage remains very low at 3% [30] and many women still pay out-of-pocket for antenatal services. Similarly, in the absence of a national program for free or subsidized antiviral drugs, the cost of treatment of HBV has remained prohibitive [31].

A common view also expressed by the respondents was the HBV knowledge gap. Indeed studies have reported poor knowledge of HBV among pregnant women [15, 32] and health workers [33–35] in Nigeria. Notwithstanding the high burden, HBV has received limited awareness campaigns compared with an infectious disease such as HIV. This may be responsible for the ignorance among pregnant women noted by the respondents. Myths and misconceptions from lack of knowledge may affect the demand and uptake of HBV screening test. Poor awareness may also be associated with low-risk perception and the perceived need for screening by pregnant women and health workers. While there is a need for a mass campaign about HBV screening, incorporating it into routine antenatal counselling may improve knowledge and facilitate the uptake of screening among pregnant women. This will also require strengthening the capacity of the health workers to provide such services.

Our findings, although limited to health workers, are consistent with previous studies in low-income countries that have identified lack of test kits, high cost of testing and treatment, limited public awareness, and a paucity of skilled health providers as barriers to HBV screening among pregnant women and other populations [17, 18, 36–38].This suggests that these challenges are not unique to Nigeria, and it calls for a regional effort to ensuring access to low-cost testing and treatment. Governments should also begin to prioritize HBV testing and allocate more resources to address the burden. In maximizing resources, the HIV programs in many countries can be leveraged for the prevention and control of vertical transmission of HBV [39].

To the best of our knowledge, our study will be among the first to present the barriers to HBV screening of pregnant women from the perspective of health workers in Nigeria. Nonetheless, the representativeness of our findings is limited by the number of included states. The survey was self-administered and the health workers’ responses in our study might have been affected by their ability to express their views in writing and personal biases. While the views of the respondents might truly reflect barriers at provider and health system levels, they might not fully reflect the patient-level barriers for the pregnant women. We recommend further qualitative studies on the barriers to HBV screening tests, particularly from the perspectives of pregnant women. Implementation science research is also needed to test and identify effective strategies for improving HBV screening rates. Finally, our findings highlight the need for a national service availability and readiness assessment survey for HBV prevention and treatment services.

## Conclusions

The findings from this study suggest that HBV screening of pregnant women attending PHCs in Nigeria is affected by multilevel barriers. To eliminate perinatal transmission of HBV in Nigeria, the highlighted factors at the patient, provider and health system levels must be addressed through effective and sustainable interventions. These should include interventions targeted at improving the availability of HBV test kits and other essential commodities, strengthening the capacity of health providers, making the HBV vaccine and treatment more affordable, and improving HBV awareness.

## Data Availability

All data generated or analyzed are available from the corresponding author on reasonable request.

## Abbreviations

CHEW: Community health extension worker
CHO: Community health officer
JCHEW: Junior community health extension worker
HBV: Hepatitis B virus
LGA: Local government area
PHC: Primary healthcare center
PMTCT: Prevention of mother-to-child transmission
